# Supplementary material for: Chromothripsis during telomere crisis is independent of NHEJ, and consistent with a replicative origin
Source: Genome Res. 2019 May;29(5):737–49. doi: 10.1101/gr.240705.118 (PMC6499312; doi:10.1101/gr.240705.118)
Supplement: Supplemental Material [file supp_gr.240705.118_Supplemental_file_1.zip › contigs/annotated_contigs/DB90/contig.8.DB90_length_620_mean_cov_14.0322580645.docx]

**DB90_length_620_mean_cov_14.0322580645**

```
CACTCTGGCACACATCGAACCTTCTTTCTGTAAACCTCTATACACATATGAAACTCAGAATGTCCTGGTAATGCTTAGCTGTGAGGAAT
 >chr8:54506326-54506573 + E=7e-134 p=3e-03
CTCATCTTCCCCTCAGTAAGCCAGTTCTCTCAAAAGGCAGCGACCAGTCATACCGATTCTTATACCCCTTGAGTATCTCCCATGGAACA

TGGCCTGGTGCTGAACCTGTGAATTTGCAGTGAACACACAACACACAAGACTCGCCACATCAC|ATACAC|ACCACACACCCCATATAT
                                                                >chr21:44888527-4488855
ACACA|CA|ATATGCACATACACCACATACACACCACACAC|CCCA|TATATATACACAATACACACACACCACATAGA|CATCGTATA
6 - E=1e-02 p=0e+00   >chr22:48938138-48938170 - E=2e-01 p=0e+00  >chr21:44260398-44
TACACACAAT|ACACACACCACATACACACCGTATATACACACA|AT|ACACACACCACATACACAACACACATCACACCAAATATACA
260430 - E=2e-01 p=0e+00  >chr12:56366928-56366961 + E=4e-02 p=0e+00  >chr13:31417146-
CA|ACACACAAGACACATCACACCACATACACACCACACAAGGACAAGACACATCA|CACACACCATATACACACCACACAAGACACAC
31417188 - E=3e-03 p=0e+00                              >chr11:2208436-2208473 + E=2e-0
CACAC|CAACAACCACAAAAGACACATCATACTACATACACACCACACATACCATATATAT|ACAAAATACACACACATCATATATATA
1 p=0e+00                                                    >chr15:55597038-55597066 -
T|GAAATACACA E=4e-02
```
